# Great Offset Loading Influences Core and Bench Press Peak Prime Mover’s Activity in Trained Athletes

**DOI:** 10.3390/jfmk10020180

**Published:** 2025-05-16

**Authors:** Bernat Buscà, Jordi Arboix-Alió, Clàudia Baraut, Adrià Arboix, Joan Aguilera-Castells

**Affiliations:** 1 Faculty of Psychology, Education Sciences and Sport Blanquerna, Ramon Llull University, 08022 Barcelona, Spain; bernatbs@blanquerna.url.edu (B.B.); claudiabg2@blanquerna.url.edu (C.B.); joanac1@blanquerna.url.edu (J.A.-C.); 2 Faculty of Health Sciences Blanquerna, Ramon Llull University, 08025 Barcelona, Spain; 3 Sport Performance Area, FC Barcelona, 08970 Barcelona, Spain; 4 Cerebrovascular Division, Department of Neurology, Hospital Universitari del Sagrat Cor, Universitat de Barcelona, 08029 Barcelona, Spain; aarboix@hscor.com

**Keywords:** asymmetric training, symmetry loading, resistance exercises, electromyography, instability

## Abstract

**Objectives**: This study aimed to compare the acute responses of the muscular activity of primary movers during bench press execution under asymmetric loads (25%, 50%, and 75%). **Methods**: The study included 30 resistance-trained males (n = 25, age = 22.73 ± 3.44 years, height= 1.77 ± 0.06 m, body mass= 76.77 ± 9.28 kg) and females (n = 5, age = 22.5 ± 1.19 years, height = 1.63 ± 0.04 m, body mass = 56.78 ± 2.90 kg). We assessed the two portions of the dominant pectoralis major, triceps brachii, anterior deltoid, and both external oblique peak activities (sEMG) during concentric and eccentric phases. We performed a repeated-measures design to establish the differences between muscle activity, barbell center of mass acceleration, and OMNI-Perceived Exertion Scale for Resistance Exercise (OMNI-RES) in a bench press under seven different conditions. **Results**: The linear mixed model showed a significant fixed effect for exercise condition for muscles (*p* < 0.001) in the concentric and eccentric phases. We found significantly higher clavicularis (*d* = 0.54; *d* = 1.15) and sternalis (*d* = 0.38; *d* = 0.86) pectoralis major activation of the dominant side under high (50% and 75%), non-dominant-side, de-loaded conditions in the eccentric phase (*p* < 0.001), with large effects. Contralateral core muscles (external oblique) of the dominant and non-dominant sides were significantly (*p* < 0.001) highly activated under all asymmetric conditions in the concentric phase (from *d* = 0.89 to *d* = 2.30). **Conclusions**: The asymmetric load bench press provoked a higher pectoralis major activation on the loaded side when de-loading the other side. The contralateral external oblique doubles the muscle activity in the most asymmetric conditions.

## 1. Introduction

New challenging and combined strength training techniques have become an increasingly popular approach for improving athletic performance. The combined activation of prime movers, antagonists, and stabilizing muscles supports the use of complex exercises that introduce instability and uncertain muscular demands [1,2]. Beyond the use of unstable surfaces and other challenging displays to perturb posture and dynamic balance, unstable training environments can especially benefit the upper body muscles involved in precise, powerful, and complex actions. Indeed, neuromuscular training under such conditions represents an enriched stimulus for the somatosensory, vestibular, and visual systems [3], thus creating perturbations, increasing co-contractile activity, and enhancing the role of antagonists to mitigate the uncertainty produced by instability [4]. In this vein, surface electromyography (sEMG) serves as a valuable and accessible tool for quantifying muscle activation patterns and assessing the degree of muscular involvement during specific exercises, providing insights into neuromuscular function and coordination. This approach assesses the electrical activity generated by the depolarization of muscle fiber membranes during motor unit activation, providing a non-invasive measure of the timing, intensity, and pattern of muscle recruitment, which reflects the summation of action potentials from multiple motor units beneath the electrode site [5]. Furthermore, the signal normalization allows comparing the muscular involvement of the different variations of a given task, thus re-scaling the electrical signal to a percentage of selected value [6]. Beyond the muscle activity and forces produced in a certain task, effort perception (OMNI-RES) has been suggested as a valid approach to assess the intensity (blood lactate levels) and total load lifted in resistance training [7]. Thus, using a 0 to 10 scale, both male and female athletes can provide valuable, subjective, and valid data about the effort perceived, thus comparing different task executions under several stability conditions.

Within the context of upper-body strength training, instability is frequently enhanced through the implementation of unilateral exercise protocols [8,9], unstable support [10,11], suspension training [12,13], and the use of unstable/unbalanced loads [14]. In the context of resistance training, bench press is one of the most frequently used exercises to improve upper-body strength and power among athletes and fitness enthusiasts [15]. However, when an athlete with upper-limb asymmetry performs a barbell bench press, the barbell’s center of mass can shift laterally towards the stronger side, resulting in uneven lifting. Over time, this may exacerbate strength differences between sides and lead to overloading the dominant side [16]. Therefore, performing the bench press with unstable loads could be an effective strategy to alter muscle activity and to mitigate asymmetries caused by sports requirements [17]. Among these types of practices, performing exercises with an asymmetrical external load position (offset training) has been infrequently studied [18]. Lifting a one-side-de-loaded barbell requires the athlete to lift weights in a controlled manner without wiggling or shifting, thus forcing the athlete to synchronize movements [18]. In this vein, the grip position is one of the main keys to produce the desired effect of asymmetric loads. It is well known that the lateral forces exerted during a bench press exercise are influenced by several biomechanical issues including grip type, grip position on the barbell, back curvature, feet position, and elbow aperture [19]. For this reason, a symmetric grip position guarantees maintaining elbow and shoulder angles in a balanced sticking region [20]. Thus, the side’s load difference leads to the production of an unstable effect that neuromuscular system needs to neutralize.

To the best of our knowledge, three recent studies have analyzed the influence of performing weight exercises with asymmetric loads on muscle activity. Jarosz et al. compared peak muscle activity in upper body muscles between symmetric and asymmetric loads (2.5%, 5%, and 7.5% differences) during the flat bench press exercise at 70% 1-RM [18]. The results indicated that the asymmetrically loaded bench press led to significantly higher muscle activity on the loaded side. Similarly, Saeterbakken, Solstad, Behm et al. compared muscle activity in the bench press between symmetric, 5%, and 10% asymmetric loads, showing higher external oblique activity during both asymmetrical conditions than during the symmetric condition [16]. Most recently, Ottinger et al. compared the ground reaction forces and muscle activity of the leg and core muscles during a traditional and asymmetric loading (10% difference) parallel squat [21].

As mentioned, the recent literature on the effect of asymmetric bench press exercise on muscle activity is limited, reaching up to 10% unbalanced barbells. Therefore, the main aim of the present study was to compare the changes in activity on the dominant side of the upper body and core muscles during the execution of a flat bench press under different asymmetric conditions, with greater load differences between sides. We hypothesized that muscle activity would be greater on the non-de-loaded side and lower on the de-loaded side of the prime movers (pectoralis clavicularis, pectoralis sternalis, anterior deltoid, and triceps brachii) compared to the symmetric condition. Additionally, we hypothesized that both external obliques would show increased activation in all asymmetric conditions. Furthermore, we expected that greater asymmetrical loads would lead to a greater increase in barbell center of mass acceleration (BCMA) and OMNI Perceived Exertion Scale for Resistance Exercise.

## 2. Materials and Methods

### 2.1. Participants

We recruited thirty resistance-trained athletes, comprising male (age = 22.73 ± 3.44 years, range: 18–31 years; height= 1.77 ± 0.06 m, body mass= 76.77 ± 9.28 kg) and female (age = 22.5 ± 1.19 years, range: 21–24 years; height = 1.63 ± 0.04 m, body mass = 56.78 ± 2.90 kg) athletes. All participants had at least five years of strength training experience and were engaged in 3–4 strength training sessions per week; those who had a history of cardiovascular, metabolic, or neurological diseases or injuries; were unable to perform a bench press at over 80% of their 1-RM under controlled conditions; had undergone upper-limb orthopedic surgery within the previous two years; or were using medications known to affect muscle function—such as corticosteroids, muscle relaxants, or stimulants—were subsequently excluded from the study. Before the participation in the study, written informed consent was obtained from all participants. This study was approved on 2 April 2024 by the Ethics and Research Committee Board at the Blanquerna Faculty of Psychology and Educational and Sport Sciences of Ramon Llull University of Barcelona (reference number 1819005D). All protocols conducted in this study complied with the requirements of the Declaration of Helsinki (revised in Fortaleza, Brazil, 2013).

### 2.2. Study Design

A repeated-measures design was used to establish the effects of different asymmetric loads on muscle activity, BCMA, and OMNI-RES. Participants performed a resistance bench press under seven conditions (symmetric loads, 25% dominant side de-loaded, 50% dominant side de-loaded, 75% dominant side de-loaded, 25% non-dominant side de-loaded, 50% non-dominant side de-loaded, and 75% non-dominant side de-loaded). The symmetric load was 60% 1-RM for each participant, and the other conditions randomly de-loaded were 25%, 50%, and 75% in the dominant and non-dominant sides, respectively. Across the seven conditions, sEMG was used to measure muscle activity in the dominant upper limb on the following muscles: (1) pectoralis clavicularis (PC); (2) pectoralis sternalis (PS); (3) external oblique of the dominant side (EO-D); (4) external oblique of the non-dominant side (EO-ND); (5) anterior deltoid (AD); and (6) triceps brachii (TB). A triaxial accelerometer was placed on the barbell to measure the BCMA. OMNI-RES was collected at the end of each condition.

### 2.3. Procedures

**Figure 1:**
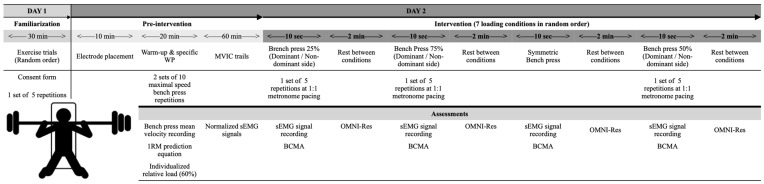
Study design chart.

The study was conducted in two sessions, both performed at the same time in the morning and one day apart. During the first session, the subjects were familiarized with asymmetric loads, attempting all conditions in random order. In the second session, we collected the sEMG, BCMA, and OMNI-RES of the participants when performing bench press exercises under all conditions. In this session, subjects performed 10 min of general warm-up, dynamic stretches, and joint mobility of the upper limb and warm-up bench press series. During the specific warm-up, two sets of 10 maximal-speed bench press repetitions were performed and controlled with a linear positional transducer (Chronojump-Boscosystem; Barcelona, Spain). We used the assessed mean velocity to calculate the 1-RM using a predictive equation [22]. This is a popular, valid, and time-beneficial procedure to individualize resistance training loads. After 1-RM calculation, we individualized the relative load of 60% of the 1-RM for bench press and outfitted the participants with surface electrodes. Previously, the participants’ skin was prepared to place the electrodes on the dominant side of the upper body, specifically on the EO-D, EO-ND, AD, and TB following the SENIAM Project [23], and on the PC and PS following Criswell recommendations [5]. All participants were encouraged to avoid any high-intensity physical activity or training session for 24 h before the testing session, and they consumed no food, drinks, or stimulants 3–4 h before. After a 3 min rest, the participants executed a maximal voluntary isometric contraction (MVIC) test for all recorded muscles according to the Konrad [6] protocol. Then, the participants completed the bench press conditions using a fitness barbell (total length: 1.8 m; interior length: 1.1 m; weight: 10 kg) in a random order. For instance, the loads of the 50% de-loaded condition for a participant weighing 100 kg in 1-RM were as follows: 10 kg corresponding to the barbell, 25 kg on the loaded side, and 12.5 kg on the de-loaded side. Participants were asked to maintain a symmetric grip location throughout the testing conditions. This is a crucial matter because displacing the grip location to one side probably mitigates part of the muscular effort to maintain a balanced lifting execution. Thus, for each exercise condition, subjects completed a set of five repetitions at 60% 1-RM (symmetric condition) and the corresponding de-loadings (asymmetric conditions) at a pace rate of 1:1, controlled by a digital metronome at 60 beats per minute. We assessed vertical displacement under all exercise conditions using a linear positional transducer (WSB 16K-200; ASM Inc., Moosinning, Germany) attached to the tether of the transducer at the barbell. The participants performed all bench press conditions while maintaining posture as consistently as possible. The range was from the chest to full elbow extension. The participant’s feet were placed flat on the floor over normalized marks (90° of knee flexion), with the toes pointing forward. The shoulders and lower back were all the time in contact with the bench (Figure 1). We randomized the order of bench press conditions and allowed 2 min of rest.

### 2.4. Surface Electromyography Signals

Before collecting the sEMG signals, we placed bipolar surface electromyography electrodes (Biopac EL504 disposable Ag-AgCl) on the muscles previously mentioned. We recorded and analyzed the sEMG signal during each repetition using a six-channel BIOPAC MP-150 data acquisition system at a sampling rate of 1.0 kHz and AcqKnowledge 4.2 software (BIOPAC System, Inc., Goleta, CA, USA). We then bandpass filtered the sEMG signal of all analyzed muscles at 10–500 Hz, utilizing a fourth-order Butterworth filter, and acquired the root mean square (RMS). In order to normalize the sEMG signal, we expressed the RMS as %MVIC using the MVIC values recorded for each muscle in the MVIC trials as a baseline [24]. The categorization of muscle activation was as follows: very high (>60% MVIC), high (41–60% MVIC), moderate (21–40% MVIC), and low (<21% MVIC). We analyzed the peak sEMG of the middle three repetitions under all exercise condition sets during the concentric and eccentric phases. We used the average peak sEMG value from the middle of the three repetitions of each exercise condition for the data analyses.

### 2.5. Barbell Center of Mass Acceleration

We measured BCMA using a tri-axial accelerometer TSD109F (BIOPAC System, Inc., Goleta, CA, USA) with a sample rate of 2.0 kHz, sensitivity of 40 mV/g, and range of ±50 g. We placed the calibrated accelerometer at the midpoint of the bar’s total length. We also bandpass filtered the acceleration signals on the vertical axis fixed at 0.5 Hz (low) and 20 Hz (high), and then integrated them with an RMS. Subsequently, we considered the sum of amplitudes in the entire phase to analyze the magnitude of the amplitudes in the middle three repetitions and calculated the average [25].

### 2.6. OMNI-Res

We collected OMNI-Res to rate the perceived exertion of the subjects for each condition. During the familiarization session, we instructed the participants to follow the protocol of Robertson et al. [7]. After completion of each exercise condition, we asked the participants to indicate their OMNI-Res rating and calculated the average scores for each condition.

### 2.7. Statistical Analysis

We used Jamovi Statistical Software (Jamovi Project, 2023; version 2.3) to analyze the statistical data. A preliminary power analysis for a linear mixed model was performed using General Linear Mixed Model Power and Sample Size software (GLIMMPSE; version 3.0.0) to determine an adequate sample size. By establishing the alpha level set at 0.05, with a power of 0.95, it was determined that 30 participants would be needed. After verification of data distribution normality, we carried out a linear mixed model to assess the acute effects of bench press condition, as a fixed effect, on muscle peak sEMG (PC, PS, EO-D, EO-ND, AD, and TB), BCMA, and OMNI-Res as dependent variables, and participants as the random effects. In case of a significant fixed effect (*p* < 0.05), we performed post hoc comparisons using Bonferroni correction. In the previous model, we evaluated the significance of the fixed effects related to the outcome variable by using the Wald test. After model validation, we examined the residuals from the final models to ensure that they met the assumptions of normality, homogeneity, and independence. Subsequently, we assessed the normality of the residuals using a normal Q-Q plot. Additionally, we also calculated the Cohen’s *d* effect size [26], and interpreted as <0.2 = trivial; 0.2–0.6 = small; 0.6–1.2 = moderate; 1.2–2.0 = large; and >2.0 = very large [27]. All statistical significance was set at *p* < 0.05.

## 3. Results

The linear mixed model showed a significant fixed effect for exercise condition for PC (F_(6,168)_ = 16.95; *p* < 0.001), PS (F_(6,168)_ = 55.12; *p* < 0.001), EO-D (F_(6,168)_ = 17.90; *p* < 0.001), EO-ND (F_(6,168)_ = 35.09; *p* < 0.001), AD (F_(6,168)_ = 29.18; *p* < 0.001), and TB (F_(6,168)_ = 9.30; *p* < 0.001) for the concentric phase. Fixed effects for the eccentric phase were also significant for PC (F_(6,168)_ = 51.90; *p* < 0.001), PS (F_(6,168)_ = 70.17; *p* < 0.001), EO-D (F_(6,168)_ = 19.50; *p* < 0.001), EO-ND (F_(6,168)_ = 34.41; *p* < 0.001), AD (F_(6,168)_ = 63.65; *p* < 0.001), and TB (F_(6,168)_ = 24.45; *p* < 0.001).

**Figure 2:**
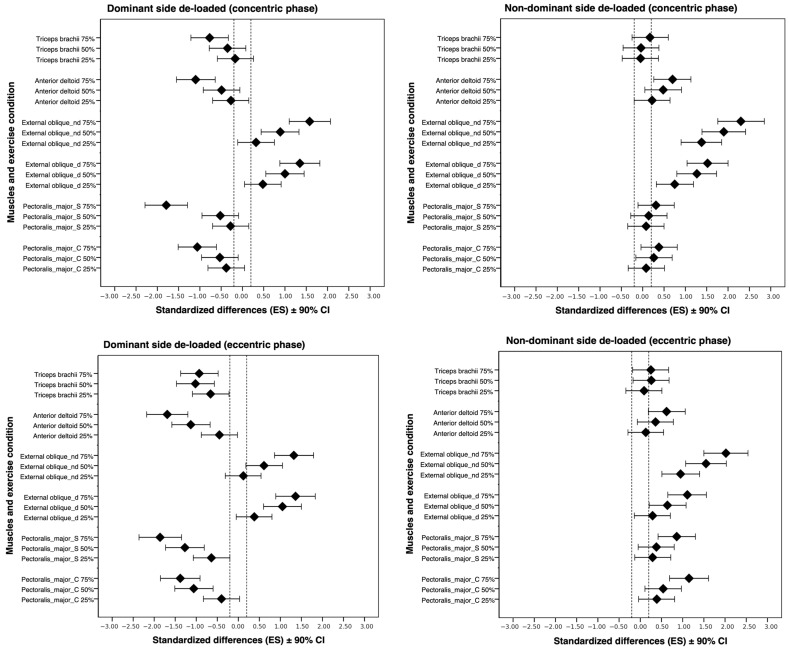
Effects of asymmetric de-loadings on sEMG activity for each analyzed muscle. Bars represent the 90% confidence interval for the effects of conditions with respect to the symmetric condition. Dotted lines represent the smallest substantial threshold. C indicates clavicular portion; S, sternal portion, d, dominant; nd, non-dominant.

Table 1 shows the normalized sEMG (% MVIC) mean ± standard error of the measured values for each analyzed muscle under different conditions for the concentric and eccentric phases. Figure 2 shows the Cohen’s *d* effect size with 90% confidence intervals for the concentric and eccentric phases.

**Figure 3:**
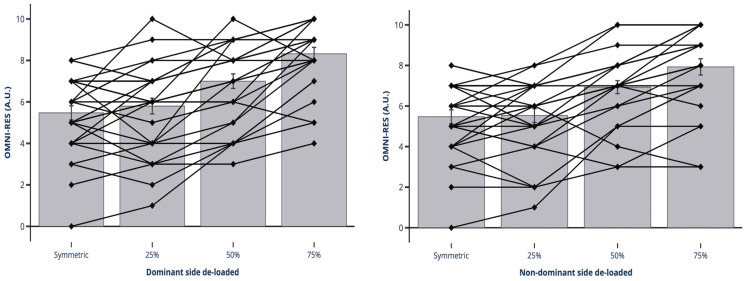
Mean and individual OMNI-Perceived Exertion Scale for Resistance Exercise for dominant and non-dominant de-loaded conditions A.U. indicates arbitrary units.

The OMNI-Res showed a significant fixed effect in the exercise condition (F_(6,169)_ = 35.40; *p* < 0.001). All conditions significantly differed with respect to the others (*p* < 0.001), except for the conditions de-loading the same amount of load (dominant vs. non-dominant side) and the symmetric condition with 25% de-loading for both the dominant and non-dominant sides (*p* = 1). Figure 3 shows the individual perception of exercise difficulty, the mean for each condition, and the standard error of the measure for the dominant and non-dominant side de-loaded condition.

Additionally, no significant fixed effect was found on BCMA (F_(6,138)_ = 1.40; *p* = 0.219) for exercise conditions.

## 4. Discussion

The primary objective of this study was to examine the variations in muscle activity on the dominant side of the upper body (pectoralis clavicularis, pectoralis sternalis, anterior deltoid, and triceps brachii) and core (external oblique) during the performance of the flat bench press under varying asymmetric loading conditions. Asymmetric loads influence the muscle activity of the prime movers in bench press exercises. Thus, de-loading 75% of the non-dominant side of the barbell, dominant PC, and PS reached a higher peak of activity during the eccentric phase but not during the concentric phase. Moreover, the core muscles evaluated (EO-D and EO-ND) showed increased activity in all asymmetric conditions, especially the EO-ND when the non-dominant side was de-loaded. Other findings were that all participants were able to stabilize their barbell under all conditions, with no significant differences in BCMA. Furthermore, higher de-loadings (50% and 75%) meant significantly higher OMNI-Res, but not between the same % of de-loading on the dominant and non-dominant sides.

To the best of our knowledge, only two studies have investigated this topic in bench press [16,18]. Although the authors did not report specific information, both studies presumably used an Olympic barbell (total length: 2.2 m; interior length: 1.31 m; weight: 20 kg), while the present study performed the exercise using a fitness barbell (see Section 2). This is the main reason why the magnitudes of the asymmetric loads in the present study were higher (25%, 50%, and 75% de-loadings) compared to the two aforementioned studies that used increments of 2.5%, 5%, and 7%, and decrements of 5% and 10% to obtain the offset effect, respectively. The load position on the barbell determines the torque created by the asymmetric loads. Moreover, the grip position might not have been the same in all three studies. While Jarosz et al. did not provide information about this factor [18], Saeterbakken, Solstad, Behm et al. provided a picture where one can observe the possibility of performing an asymmetric grip [16]. In the present study, we asked for a symmetric grip during exercise execution. In this vein, the grip position and the total torque have been demonstrated as important factors in determining the forces exerted in a bench press exercise [19,28]. Thus, these factors and other technique specificities, such as feet position or back curvature, might influence the muscle activity during bench press execution in the three studies. Unfortunately, the authors did not always provide detailed information to discuss such matters in depth. Nevertheless, after load individualization, it could be expected that the non-de-loaded side would exert similar forces, while the de-loaded side would produce lower forces. In the present study, the greater loading asymmetries may have amplified these force production differences, particularly under the 50% and 75% de-loading conditions. However, the only study to date that has assessed force production during asymmetrically loaded squat execution reported no significant differences between the dominant and non-dominant sides—likely due to the nature of the exercise (a standing lower-limb task) and the relatively small load offset of 5% [21]. It is therefore plausible that larger asymmetries in loading may lead to significant bilateral differences in force output.

The pectoralis major is the most demanded muscle in bench press exercises [29]. Although the two portions of the pectoralis (sternalis and clavicularis) have similar roles as a primary mover in adduction, flexion, and medial rotation of the arm at the glenohumeral joint, in a bench press exercise, the PS portion deeply contributes to extension of the flexed arm and PC to the flexion of the extended arm [30]. Such specific roles motivated us to specifically analyze their activity in the concentric and eccentric phases. Although the concentric phase clearly demanded both pectoralis portions, we found significant muscle activity increases only in the eccentric phase under the 75% non-dominant-side de-loaded condition, but not in the other de-loadings. Thus, differences appeared only during the eccentric phase when the condition was extremely challenging, even though the total load was low. In both phases, we found an expected reduction in muscle activity on the de-loaded side for both pectoralis portions under the 50% and 75% de-loaded conditions; however, we found no significant reduction under the 25% de-loaded condition. Thus, in contrast with all de-loadings tested by Saeterbakken, Solstad, Behm et al., 25% of dominant-side de-loading did not change significantly in the pectoralis portions, probably because of the setup and barbell differences between both experiments [16].

Even when the barbell was de-loaded (50% and 75%) on the non-dominant side, AD activity increased on the dominant side. Their role as a shoulder elevator helps in movement stabilization [31]. Accordingly, Saeterbakken, Solstad, Behm et al. found significant muscle activity reduction under a 10% de-loaded condition in the de-loaded side but similar activity in the dominant side when the non-dominant side was de-loaded [16]. In contrast, Jarosz et al. found that AD increased the activity on the loaded side and decreased the activity on the de-loaded side [18]. These differences might be motivated by the effect of the big force momentum created in the present study, especially in higher asymmetries, thus forcing the AD to reinforce the stabilizing role of the scapula during the adducted arm while performing the flexo-extension movement [19]. Furthermore, TB activity was similar under all conditions. Only higher de-loadings (50% and 75%) on the dominant side significantly lowered muscular demands. The lack of grip width variation and its similar role during the final phase of the lift might explain this finding [19]. In contrast to Seaterbakken, Solstad, Behm et al., we found lower TB activity in the de-loaded side, probably because of the differences in barbell velocity in our study and the load differences [16]. To maintain the ecological validity of lifting, the authors asked for maximal effort over 5-RM (approximately 85% 1-RM) loading, while we standardized a 60% 1-RM and controlled pace (60 bpm). Therefore, the final phase of the lift was more demanding on the non-dominant side to maintain the barbell centered over the sternum when the effort was maximal [16], but this did not happen at a controlled pace with lighter loads and, therefore, there were lower lateral accelerations to compensate, together with a lower velocity during the final arm extension. Nevertheless, our finding agrees with Jarosz et al., who used similar loads (70% 1 RM), although the authors did not provide specific information about the pace [18].

In accordance with previous research [16], the external oblique was significantly highly demanded under all asymmetric conditions on the contralateral side of the loaded side. The magnitudes of these reported increased activities were similar in both the movement phases. In our study, asymmetric conditions almost doubled external oblique muscle activity (between 100% and 215% in both dominant and non-dominant sides), and Saeterbakken, Solstad, Behm et al. reported even more difference in the most loaded unbalanced condition (up to 280 and 320% more in the 5% and 10% de-loaded conditions, respectively), probably because of the greater torque created by the barbell used [16]. In this respect, the role of core muscles in stabilizing body posture under unstable conditions has been widely demonstrated [32,33]. Synergistically with the internal oblique, the external oblique acts as a trunk flexor and side bend rotator. During asymmetric lifts, this muscle plays a crucial role in slightly rotating the trunk toward the contralateral side to maintain the hip position on the bench. Other authors have also observed similar greater core muscle activation in bench press exercises using swinging loads [34], fitball support [35], and dual instability [33]. These mentioned methodological approaches similarly attempt to destabilize the athlete execution, thus trying to alter the muscular coordinative patterns including the enhanced participation of core muscles. Although we observed the largest differences in the concentric phase of the largest de-loadings in the contra-lateral (de-loaded side) external oblique, the loaded side was also significantly more demanded in the 50% and 75% asymmetric conditions, especially during the concentric phase. This finding reinforces the role of both core sides in stabilizing the body on the bench when lifting asymmetric loads.

The lack of differences between conditions in the BCMA meant that all subjects were able to maintain barbell stability during the whole movement, in contrast with Buscà et al., who used a similar procedure to establish the amount of instability in different half-squat conditions [25]. In this study, the authors used a more sensitive approach to understand how center of body mass shifted in the lateral and anteroposterior axis, instead of using floor-based tools (force platforms, stabilometers, or pressure mats). In their study, the authors utilized a more sensitive method to evaluate shifts in the center of body mass along the lateral and anteroposterior axes, rather than relying on traditional floor-based tools such as force platforms, stabilometers, or pressure mats. In the present study, an accelerometer was placed directly on the barbell to capture the magnitude of micro-destabilizations required to preserve barbell equilibrium. Even with the use of this method to quantify micro-destabilizations, participants demonstrated an ability to maintain barbell stability under asymmetric loading conditions comparable to that observed under symmetric loading. Furthermore, while both upper- and lower-body resistance exercises require neuromuscular coordination, stabilizing external loads during lower-body movements such as the squat demands greater vestibular system involvement due to the upright posture and dynamic head position changes. In contrast, upper-body exercises like the bench press involve a supported position, thereby reducing the reliance on vestibular input [36]. Nevertheless, in accordance with Marshall and Murphy, we found significant differences in the most unstable conditions (50% and 75% dominant and non-dominant side de-loadings, respectively) when the subjects were asked for their effort perception (OMNI-Res) [37]. In contrast, Panza et al. found no significant differences when comparing the perceived exertion of performing a bench press on a Swiss ball instead of a flat bench [38]. This may be explained by the fear of losing load control under the most asymmetric conditions proposed in our study.

The subjects of this study were experienced strength training athletes, and were familiar with offset training. Similar populations were tested in the literature with similar findings [18]. Therefore, it is hard to extrapolate these data for high-standard athletes or recreational athletes with no experience. Although it is interesting to note that the barbell length and size largely influences the amount of asymmetry that one can display in offset training, the use of a fitness barbell makes it difficult to compare the % of de-loadings with the existing literature. In addition, our interest in studying the role of the prime bench press movers and bilateral core muscles, and the limitation of sEMG channels, made it not possible to assess the non-dominant side muscles.

The selection of the barbell is a crucial factor for establishing the amount of asymmetry as a percentage. Thus, longer and heavier barbells admit lower amounts of asymmetry and vice versa. One of the most important factors for neuromuscular and hypertrophic adaptations in sports strength and conditioning training is the volume lifted [39]. In this vein, involving core training during the execution of upper and lower-body limb exercises might be a complementary strategy to free up more time to stimulate the prime movers. Furthermore, offset training could be an appropriate practice to minimize the muscular and functional asymmetries, which can be harmful because of exacerbation of the limb differences throughout the training process [16]. Nevertheless, the fear of losing the load control must be managed by the coaches and practitioners to safely introduce offset training. Progressive de-loadings and shorter barbells might be more appropriate for beginners in such practices.

Despite the utility of these findings, it is important to acknowledge some limitations of the present study. Firstly, the use of a fitness barbell, which differs in length and weight distribution from a standard Olympic barbell, limits the generalizability of our findings and makes direct comparison with the previous literature difficult. Second, due to the limitations in sEMG channel availability, we were unable to measure all bilateral muscle activities, particularly those of the non-dominant arm. Third, our participant sample included trained individuals with prior experience in offset training, which may not reflect the responses of untrained or elite athletes. Lastly, the use of a standardized load (60% 1-RM) and controlled movement cadence may have reduced ecological validity when compared to real-world training scenarios that involve higher intensities and self-selected tempos.

Future studies should explore the neuromuscular and functional effects of offset training across different populations, including novice lifters, elite athletes, and individuals with existing muscular imbalances. Longitudinal research is also warranted to examine the chronic adaptations resulting from consistent use of asymmetric loading in strength training programs. Furthermore, future experiments should aim to analyze a broader range of muscle groups using high-channel sEMG systems, incorporate different barbell types, and test varying grip widths and tempos to better simulate real training conditions. Finally, understanding how offset training influences injury risk and performance outcomes in sport-specific contexts would provide valuable insights for practitioners and coaches.

## 5. Conclusions

In conclusion, asymmetric loads provoke a significantly higher clavicular and sternal pectoralis activation of the dominant side when one performs high (75%) de-loadings. This happens mainly during the eccentric phase of the bench press. Moreover, contralateral core muscles (external oblique) of the dominant and non-dominant side were significantly highly activated under all asymmetric conditions. Such differences reached 200% in the most asymmetric condition (75% de-loading). Even though all subjects were able to control the barbell because we found no significant differences in the BCMA, subjects reported different effort perceptions for the most asymmetric conditions (50% and 75%).

The findings of this study suggest that offset training using asymmetric loads in the bench press can be a valuable strategy to selectively increase activation of both upper-body prime movers and core stabilizers. Strength and conditioning coaches and practitioners may incorporate moderate to high asymmetries to enhance core engagement and challenge motor control without compromising barbell stability. This approach can be particularly useful for addressing unilateral weaknesses, improving muscular balance, and integrating core activation into upper-body strength exercises.

## Figures and Tables

**Table 1:** Muscle activity (mean ± SEM) for all experimental conditions.

		Dominant Side De-Loaded			Non-Dominant Side De-Loaded		
CONCENTRIC	Symmetric	25%	50%	75%	25%	50%	75%
Pectoralis-major_C	105.43 ± 5.43	93.82 ± 5.73	88.65 ± 6.18 *	74.23 ± 5.59 *	107.82 ± 5.17	113.05 ± 5.09	116.45 ± 5.35
Pectoralis_major_S	117.74 ± 7.25	106.73 ± 7.19	96.19 ± 7.93 *	54.03 ± 5.92 *	120.75 ± 6.88	123.39 ± 6.31	129.52 ± 6.78
External oblique_d	12.71 ± 1.22	21.46 ± 2.74 *	31.13 ± 3.54 *	36.34 ± 4.04 *	15.97 ± 1.17	21.07 ± 1.79 *	26.83 ± 2.54 *
External oblique_nd	12.18 ± 0.94	14.26 ± 1.40	18.23 ± 1.50	25.02 ± 1.98 *	24.42 ± 2.10 *	33.52 ± 2.75 *	38.41 ± 2.94 *
Anterior deltoid	100.23 ± 4.65	93.05 ± 4.97	86.44 ± 5.52 *	70.25 ± 5.58 *	106.51 ± 5.71	113.68 ± 5.62 *	120.19 ± 6.04 *
Tricep brachii	91.98 ± 4.63	87.78 ± 4.55	83.67 ± 4.02 *	73.81 ± 4.16 *	90.60 ± 4.82	90.96 ± 4.89	96.89 ± 5.90
**ECCENTRIC**	**Symmetric**	**25%**	**50%**	**75%**	**25%**	**50%**	**75%**
Pectoralis-major_C	66.52 ± 4.16	57.50 ± 4.03	44.41 ± 3.45 *	39.45 ± 3.03 *	75.75 ± 4.55	79.90 ± 4.81	94.24 ± 4.87 *
Pectoralis_major_S	79.88 ± 4.54	64.18 ± 4.46	51.45 ± 3.57 *	37.89 ± 3.86 *	87.53 ± 5.03	89.55 ± 4.85	106.42 ± 6.92 *
External oblique_d	10.37 ± 1.18	13.70 ± 1.95	19.98 ± 2.06 *	29.16 ± 3.53 *	12.22 ± 1.16	14.47 ± 1.15	19.40 ± 1.83 *
External oblique_nd	9.60 ± 0.95	10.28 ± 1.13	12.80 ± 0.96	17.62 ± 1.31 *	15.75 ± 1.37 *	23.40 ± 2.09 *	30.39 ± 2.61 *
Anterior deltoid	77.94 ± 5.67	65.20 ± 4.70 *	48.83 ± 3.54 *	36.22 ± 3.09 *	81.77 ± 5.02	89.03 ± 5.66	97.63 ± 6.18 *
Tricep brachii	68.06 ± 3.62	55.95 ± 3.06	49.18 ± 3.09 *	49.17 ± 4.04 *	69.82 ± 3.84	73.50 ± 4.10	74.18 ± 5.51

* indicates significantly different with symmetric condition (*p* < 0.05); C indicates clavicularis; S, sternalis; d, dominant; nd, non-dominant; and SEM, standard error of the mean.

## Data Availability

Data are available in a publicly accessible repository: The original data presented in the study are openly available at the following link: https://doi.org/10.6084/m9.figshare.28931237.v1 (accessed on 9 May 2025).
